# Discordant Perioperative Prophylaxis and Major Morbidity After Pancreatoduodenectomy in Patients Undergoing PTBD: A Culture-Based Analysis

**DOI:** 10.3390/jcm15062280

**Published:** 2026-03-17

**Authors:** Yusuf Yunus Korkmaz, Feyyaz Gungor, Ilyas Kudas, Talha Sarigoz, Birkan Bozkurt, Ozgur Bostanci, Erdem Kinaci

**Affiliations:** Department of General Surgery, Istanbul Başakşehir Çam and Sakura City Hospital, Istanbul 34480, Turkey; feyyazgngr@gmail.com (F.G.); kudasilyas@gmail.com (I.K.); sarigoztmd@gmail.com (T.S.); birkan.bozkurt@gmail.com (B.B.); ozgur.bostanci@sbu.edu.tr (O.B.); erdem.kinaci@sbu.edu.tr (E.K.)

**Keywords:** pancreatoduodenectomy, percutaneous transhepatic biliary drainage, antimicrobial resistance, antimicrobial stewardship, postoperative morbidity

## Abstract

**Background**: Patients undergoing pancreatoduodenectomy (PD) after preoperative percutaneous transhepatic biliary drainage (PTBD) frequently develop bacterobilia. While bile culture positivity has been variably linked to postoperative infections, the clinical relevance of culture data may be more closely related to perioperative antimicrobial adequacy. We aimed to evaluate whether discordant perioperative antibiotic prophylaxis—defined by mismatch between administered prophylaxis and resistance profiles from preoperative PTBD bile cultures—is independently associated with major postoperative morbidity. **Methods**: This retrospective cohort study included consecutive patients undergoing PD between January 2020 and October 2025. Major morbidity (primary endpoint) was defined as Clavien–Dindo grade ≥ III. Secondary outcomes included postoperative day 4 inflammatory markers (WBC and CRP), length of stay, and infection-related endpoints. Bile culture findings were categorized by culture status and resistance severity (no growth, low resistance, and high resistance [MDR/XDR/PDR]). Discordant prophylaxis was defined using a predefined coverage-based algorithm incorporating antimicrobial class and susceptibility profiles. Multivariable logistic regression (adjusted for age, dichotomized ASA class, and operative type) and model performance (AUC, DeLong test; Hosmer–Lemeshow calibration) were assessed. **Results**: A total of 145 patients were analyzed; preoperative bile culture status was no culture (*n* = 30), culture-negative (*n* = 59), and culture-positive (*n* = 56). Bile culture status was not associated with major morbidity (*p* = 0.406), POD4 inflammatory markers, or length of stay. Resistance severity categories were also not associated with major morbidity (15.3%, 17.4%, and 24.2% across no-growth, low-resistance, and high-resistance groups, respectively; *p* = 0.77). Discordant prophylaxis occurred in 23 patients (15.9%) and was associated with higher major morbidity compared with concordant coverage (30.4% vs. 18.0%; OR: 1.99, 95% CI: 0.69–5.36; *p* = 0.25). After adjustment, discordant prophylaxis showed a higher point estimate for major morbidity (adjusted OR: 1.84, 95% CI: 0.63–4.96; *p* = 0.24), although this did not reach statistical significance. The core clinical model showed poor discrimination (AUC 0.59); adding microbiological variables modestly increased the AUC to 0.63 without significant improvement (DeLong *p* = 0.46). Model calibration was acceptable (Hosmer–Lemeshow *p* = 0.88). **Conclusions**: In this PTBD cohort undergoing PD, bile culture positivity and resistance severity were not independently associated with major postoperative morbidity. Discordant prophylaxis was associated with a numerical increase in major morbidity; however, this finding did not reach statistical significance and should be interpreted cautiously given the limited sample size. These findings support interpreting bile culture data primarily within an antimicrobial stewardship framework to ensure adequate coverage rather than as standalone predictors of severe morbidity and warrant validation in larger prospective cohorts.

## 1. Introduction

Pancreatoduodenectomy (PD) is one of the most complex upper abdominal surgical procedures performed for the treatment of periampullary and pancreatic head tumors. Despite advances in surgical techniques and perioperative care, morbidity rates after PD are still reported to be in the range of 30–60%, and a significant proportion of these complications are of infectious etiology [1]. In particular, surgical site infection, intra-abdominal abscess, cholangitis, and pancreatic fistula increase the risk of mortality and prolong hospital stay, creating a financial burden. In this context, various studies have reported that bile contamination during surgery or postoperative drainage fluid contamination has a negative effect on complications after PD, including organ/space surgical site infections [2,3]. Preoperative bile culture provides important information for managing wound infections, identifying pathogens associated with infectious complications, and selecting preoperative antibiotic prophylaxis [4,5]. Thus, studies show that multidrug-resistant bacteria detected in bile cultures significantly increase the rate of surgical site infections [4,6]. This emphasizes the need to optimize the preoperative antibiotic prophylaxis regimen for the prevention and management of infectious complications in patients undergoing pancreatoduodenectomy, accounting for local epidemiology and potential resistance patterns.

Obstructive jaundice developing in periampullary tumors requires preoperative biliary drainage in a significant proportion of patients; for this purpose, endoscopic stent placement or drainage accompanied by percutaneous transhepatic cholangiography (PTC) is performed. However, it has long been thought that preoperative biliary drainage, especially bile contamination developing after drainage, increases infectious complications after PD [7,8]. Bacteria observed after endoscopic or percutaneous drainage have been shown to cause a 70–90% positivity in intraoperative bile cultures, and these bacteria often contain resistant enteric flora [9,10].

Although studies examining the relationship between bile microbiology and postoperative complications are increasing in the literature, most have focused on intraoperative bile cultures; data on the relationship between preoperative PTBD catheter bile cultures and morbidity remain limited. However, the hypothesis that the type and number of microorganisms (monomicrobial or polymicrobial) growing in the culture may affect the postoperative course—particularly in PTBD patients—has gained increasing attention. Indeed, the presence of high-risk or multidrug-resistant organisms has been associated with the development of major complications after PD. In this context, it is important to examine in more detail the role of bile cultures obtained via the PTBD catheter in predicting postoperative infectious complications in patients undergoing preoperative biliary drainage. In routine clinical practice, however, bile cultures are frequently used not only as prognostic markers but also to guide perioperative antibiotic prophylaxis. Whether discordance between administered prophylaxis and the resistance profile of organisms isolated from preoperative PTBD catheter bile cultures translates into clinically meaningful harm remains insufficiently explored, particularly in homogeneous PTBD cohorts.

Therefore, the primary aim of this study was to evaluate whether discordance between administered perioperative antibiotic prophylaxis and resistance profiles identified from preoperative PTBD bile cultures is independently associated with major postoperative morbidity (Clavien–Dindo ≥ III) after pancreaticoduodenectomy. Secondary objectives included assessing the relationship between bacterobilia status and resistance severity with postoperative outcomes, as well as evaluating the incremental predictive value of microbiological variables beyond baseline clinical factors.

## 2. Methodology

This study is a retrospective cohort analysis of consecutive patients who underwent pancreatoduodenectomy (PD) at our institution between January 2020 and October 2025. Sterile bile samples were obtained preoperatively from patients who underwent percutaneous transhepatic biliary drainage (PTBD); due to the retrospective design, intra-abdominal cultures and bile cultures were not uniformly obtained in all patients, and analyses were performed according to documented microbiological data. In routine clinical practice, bile cultures from PTBD catheters were obtained at the discretion of the surgical team rather than according to a fixed institutional protocol, typically during the preoperative hospitalization period for surgical preparation—within one week before surgery—and before the initiation of perioperative antibiotic prophylaxis.

Pancreatoduodenectomy (PD) was performed in all patients included in the study, and the surgical approach was either the classic Whipple procedure or the Pylorus-Sparing Whipple procedure. Demographic data, ASA score, BMI, type of operation, and postoperative follow-up parameters were obtained from electronic records.

Comorbidities were systematically classified based on cardiovascular, endocrine/metabolic, respiratory, neurological, psychiatric, renal, rheumatological, urological, and oncological systems, and the total comorbidity burden was calculated for each patient. Bile and abdominal culture samples were processed in aerobic and anaerobic environments; bacterial species identification was performed using the laboratory’s standard methods. Microbiological evaluation was performed within the framework of groups known to be clinically significant, and organisms were examined under the headings Enterococcus spp., Pseudomonas/Acinetobacter spp., Gram-negative flora, Gram-positive flora, and polymicrobial flora.

In this study, bacterobilia was defined as the presence of bacterial growth in bile culture samples. The terms bile culture positivity and bile colonization were used to describe microbiologically confirmed bacterial presence in bile obtained from PTBD catheters, regardless of whether clinical infection was present.

For analytical purposes, patients were first categorized according to bile culture availability (culture obtained vs. not obtained) in order to evaluate the potential impact of culture acquisition on clinical decision-making and outcomes. Among patients with available bile cultures, microbiological findings were hierarchically classified to reflect increasing antimicrobial resistance severity. This stepwise classification included the following categories: (i) no microbial growth, (ii) microbial growth without antimicrobial resistance, (iii) resistant isolates not fulfilling multidrug-resistant (MDR) criteria, (iv) multidrug-resistant (MDR) isolates, (v) extensively drug-resistant (XDR) isolates, and (vi) pandrug-resistant (PDR) isolates, using interim standard definitions for acquired resistance. For outcome analyses, these categories were further consolidated into three clinically meaningful groups: no growth, low resistance (including non-resistant and non-MDR isolates), and high resistance (including MDR, XDR, and PDR isolates), in order to ensure adequate statistical power [11].

In cases with polymicrobial growth, microbiological severity was determined according to the isolate with the highest resistance profile. This hierarchical classification was defined as an ordinal microbiological severity variable and was used in subsequent analyses to evaluate the potential graded effect of increasing resistance burden on postoperative clinical outcomes.

In addition to microbiological severity classification, a coverage-based prophylaxis adequacy variable was defined. Perioperative prophylaxis regimens were extracted from medical records and categorized according to antimicrobial class. Discordant prophylaxis was defined as administration of an antibiotic regimen lacking in vitro coverage against at least one clinically significant isolate identified in the preoperative PTBD bile culture, based on documented resistance profiles. Concordant prophylaxis was defined as coverage consistent with susceptibility testing results. This variable was incorporated into multivariable models to evaluate its independent association with major postoperative morbidity.

Coverage-based prophylaxis adequacy was evaluated using a predefined algorithm based on antimicrobial susceptibility profiles obtained from preoperative PTBD bile cultures. Perioperative antibiotic prophylaxis regimens documented in the medical records included ampicillin–sulbactam, second-generation cephalosporins, third-generation cephalosporins, ciprofloxacin, piperacillin–tazobactam, and in selected cases combination therapy including metronidazole.

For each patient with an available bile culture, antimicrobial susceptibility results were reviewed. Prophylaxis was classified as concordant when the administered antibiotic regimen provided in vitro coverage against all clinically significant isolates identified in the bile culture. In cases of polymicrobial growth, coverage adequacy was determined according to the isolate with the highest resistance profile, reflecting the organism most likely to remain untreated by the prophylactic regimen.

Conversely, discordant prophylaxis was defined as administration of an antibiotic regimen lacking in vitro activity against at least one clinically relevant organism identified in the bile culture. When antimicrobial resistance was not explicitly reported in the microbiology record, isolates were assumed susceptible according to standard selective susceptibility reporting practices.

Postoperative complications were graded according to the Clavien–Dindo classification, which is widely used for standardization in the surgical field [12]. For all patients, Clavien–Dindo complication grades were converted into numeric values ranging from 0 to 5. Complications were analyzed using two complementary approaches: (i) a dichotomous analysis, in which major complications were defined as Clavien–Dindo grade ≥ III and used as the primary endpoint; and (ii) an ordinal analysis, in which the Clavien–Dindo grade (0–5) was treated as an ordered variable reflecting complication severity. Secondary endpoints included postoperative day 4 inflammatory markers (white blood cell count and C-reactive protein levels), length of hospital stay, and infection-related clinical outcomes. Infection-focused outcomes comprised surgical site infection, intra-abdominal abscess, sepsis, prolonged antibiotic therapy requirement, and antifungal treatment requirement, and were analyzed as biologically relevant endpoints related to microbiological findings.

Continuous variables are presented as medians (interquartile range, IQR) and were compared using the Mann–Whitney U test or Kruskal–Wallis test, as appropriate. Categorical variables were analyzed using Fisher’s exact test. To evaluate factors associated with major postoperative complications (Clavien–Dindo ≥ III), multivariable logistic regression models were constructed using a predefined hierarchical modeling strategy. A core clinical model including age, dichotomized ASA classification (ASA 1–2 vs. ASA 3–4), and operative type was first developed. Microbiological variables (bile culture status and resistance severity) were subsequently added to assess their incremental contribution. In a separate model, a coverage-based prophylaxis adequacy variable (discordant vs. concordant prophylaxis) was incorporated to evaluate its independent association with major morbidity. Adjusted odds ratios (OR) with 95% confidence intervals (CI) were reported. Model discrimination was assessed using the area under the receiver operating characteristic curve (AUC), and comparison between nested models was performed using DeLong’s test. Model calibration was evaluated using the Hosmer–Lemeshow goodness-of-fit test. All analyses were performed using R software (version 4.3.1; R Foundation for Statistical Computing, Vienna, Austria). The selection of variables for the multivariable model was based on clinical relevance and previously reported risk factors after pancreaticoduodenectomy rather than data-driven variable selection. Given the limited number of outcome events, the model was intentionally restricted to a small set of clinically relevant predictors in order to reduce the risk of model overfitting. A two-sided *p*-value < 0.05 was considered statistically significant. Missing data were handled using complete-case analysis based on available data for each variable. No imputation procedures were performed because of the retrospective nature of the dataset.

This study was approved by the Scientific Research Ethics Committee No. 2 of Başakşehir Çam and Sakura City Hospital (Protocol No. KAEK/20.08.2025.291) on 9 September 2025 and was conducted in accordance with the principles of the Declaration of Helsinki. Due to the retrospective design of this study, the requirement for written informed consent was waived by the Ethics Committee.

## 3. Results

A total of 145 patients who underwent pancreaticoduodenectomy were included in the analysis. Preoperative bile culture status was categorized into three groups: no culture obtained (*n* = 30), culture-negative (*n* = 59), and culture-positive (*n* = 56). Baseline characteristics, including body mass index (BMI), ASA score, and comorbidity burden, were comparable across the three groups (Table 1).

Postoperative outcomes according to preoperative bile culture status are summarized in Table 2. No significant differences were observed among the three groups in terms of major postoperative complications (Clavien–Dindo grade ≥ III) (*p* = 0.406). Similarly, surgical site infection rates, length of hospital stay, and postoperative day 4 (POD4) inflammatory markers, including white blood cell count (WBC) and C-reactive protein (CRP), did not differ significantly between groups (all *p* > 0.05).

BMI values were also comparable across bile culture categories (*p* = 0.47). The requirement for prolonged antibiotic therapy did not differ significantly according to preoperative bile culture status (*p* = 0.77). These findings indicate that preoperative bile culture status—whether no culture was obtained, culture-negative, or culture-positive—was not associated with major morbidity, early postoperative inflammatory response, or length of hospital stay. The most frequently isolated microorganisms were *Escherichia coli*, *Klebsiella* spp., *Citrobacter* spp., *Enterococcus* spp., and *Staphylococcus* spp.

Patients were further stratified according to intra-abdominal culture status as no culture obtained (*n* = 52), culture-negative (*n* = 30), or culture-positive (*n* = 63). Postoperative outcomes according to intra-abdominal culture status are presented in Table 3. In cultures with growth, the most frequently isolated organisms were *Escherichia coli*, *Klebsiella* spp., *Streptococcus* spp., *Staphylococcus* spp., and *Enterococcus* spp.

The rate of major postoperative complications (Clavien–Dindo grade ≥ III) did not differ significantly among the three groups (*p* = 0.281). Likewise, no significant differences were observed in surgical site infection rates, POD4 WBC, or POD4 CRP levels (all *p* > 0.05).

However, the need for prolonged antibiotic therapy was significantly higher in patients with positive intra-abdominal cultures compared with those without culture growth or without culture sampling (*p* = 0.026). Despite this increase in antibiotic use, intra-abdominal culture positivity was not associated with increased major morbidity or heightened postoperative inflammatory response.

Postoperative outcomes according to the microbiological severity of bile cultures are presented in Table 4. Patients were stratified into three groups based on bile culture findings: no growth (*n* = 59), low resistance (*n* = 23), and high resistance (MDR/XDR/PDR) (*n* = 33). The rate of major postoperative complications (Clavien–Dindo grade ≥ III) did not differ significantly among the three groups (15.3%, 17.4%, and 24.2%, respectively; *p* = 0.77). Similarly, no significant differences were observed in the incidence of surgical site infection (*p* = 0.77) or sepsis (*p* = 0.92) across microbiological severity categories. The requirement for prolonged antibiotic therapy was also comparable between groups (*p* = 0.81). These findings indicate that increasing microbiological resistance severity in bile cultures was not associated with major postoperative morbidity or infection-related clinical outcomes.

A coverage-based analysis evaluating prophylaxis adequacy demonstrated that major morbidity occurred in 30.4% of patients receiving discordant prophylaxis compared with 18.0% among those receiving concordant coverage (OR: 1.99, 95% CI: 0.69–5.36; Fisher’s exact *p* = 0.25). Although this difference did not reach statistical significance, the absolute risk increase of 12.4% suggests a clinically relevant signal. In multivariable logistic regression adjusted for age, dichotomized ASA classification, and operative type, discordant prophylaxis remained associated with increased major morbidity (adjusted OR: 1.84, 95% CI: 0.63–4.96; *p* = 0.24). The core clinical model demonstrated poor discrimination for major morbidity (AUC: 0.59). Addition of microbiological variables modestly increased the AUC to 0.63; however, this improvement was not statistically significant (DeLong test *p* = 0.46). The Hosmer–Lemeshow goodness-of-fit test indicated acceptable model calibration (*p* = 0.88).

A sensitivity analysis was performed to evaluate whether the presence of extensively drug-resistant (XDR) or pandrug-resistant (PDR) bile isolates modified postoperative outcomes among patients with positive intra-abdominal cultures. Patients with concomitant intra-abdominal culture positivity and XDR/PDR bile isolates (*n* = 11) were compared with all other patients (*n* = 134), as shown in Table 5.

In this subgroup analysis, no significant differences were observed between groups with respect to major postoperative complications, prolonged antibiotic therapy, or POD4 inflammatory markers (WBC and CRP) (all *p* > 0.05). These findings suggest that even in the presence of both intra-abdominal culture positivity and a high antimicrobial resistance burden, postoperative major morbidity and early inflammatory response were not adversely affected.

This sensitivity analysis compares patients with positive intra-abdominal cultures and concomitant XDR or PDR bile isolates with all other patients. Major complications were defined as Clavien–Dindo grade ≥ III. To further evaluate whether antibiotic–pathogen mismatch influenced clinical outcomes, a coverage-based prophylaxis adequacy analysis was performed. In this analysis, isolates were assumed susceptible when resistance was not explicitly reported, reflecting routine selective resistance reporting practices. Using a predefined coverage-based algorithm incorporating antibiotic class and resistance profiling, discordant prophylaxis was identified in 23 patients (15.9%), while 122 patients (84.1%) received concordant coverage.

Major postoperative morbidity (Clavien–Dindo ≥ III) occurred in 7 of 23 patients (30.4%) with discordant prophylaxis compared to 22 of 122 patients (18.0%) receiving concordant coverage (OR: 1.99, 95% CI: 0.69–5.36; Fisher’s exact *p* = 0.25).

In multivariable logistic regression adjusting for age, dichotomized ASA classification (ASA 1–2 vs. ASA 3–4), and operative type, discordant prophylaxis showed a higher point estimate for major morbidity (adjusted OR: 1.84, 95% CI: 063–4.96; *p* = 0.24), although this difference did not reach statistical significance. Infection-related composite outcomes occurred in 25.8% of patients with discordant prophylaxis and 26.2% of those with concordant coverage (OR: 1.03, 95% CI: 0.24–4.92; *p* = 1.00), suggesting no measurable difference in infection-focused endpoints (Table 6).

## 4. Discussion

One of the notable observations of this study was the numerically higher rate of major morbidity among patients receiving discordant perioperative antibiotic prophylaxis. Although this association did not reach statistical significance after multivariable adjustment (adjusted OR: 1.84; 95% CI: 0.63–4.96), the magnitude of the observed effect and the absolute risk difference of 12.4% may represent a hypothesis-generating observation; however, given the limited statistical power and wide confidence intervals, this finding should be interpreted cautiously. These findings suggest that inadequate antimicrobial coverage may represent a potentially modifiable contributor to postoperative risk in PTBD patients, even if this cohort was underpowered to confirm the association definitively.

The determining factors of morbidity after PD have been described in detail previously. According to the current ISGPS definition, clinically significant postoperative pancreatic fistula (POPF) and other severe complications are the leading causes of mortality and prolonged hospital stay [13]. Large series and compilations have shown that soft pancreatic stiffness, small duct diameter, advanced age, high BMI, comorbidity burden, and specific tumor/pathology characteristics increase the risk of POPF and severe morbidity [14,15,16]. Analyses conducted on large national databases also support that serious complications predominantly cluster around grade B/C POPF, suggesting that surgical anatomy and host-related factors often play a central role in determining the postoperative course [14,17]. In our adjusted core clinical model, age, ASA classification, and operative type did not independently predict major morbidity. These findings suggest that severe postoperative outcomes in this cohort could not be explained solely by baseline clinical characteristics.

Biliary colonization after preoperative biliary drainage is almost the norm, and many studies have reported an association between positive bile culture and surgical site infection (SSI) in particular [18]. Systematic reviews have shown that bacterobilia is detected in approximately half of patients when intraoperative bile culture is obtained during PD; the most commonly isolated organisms are *Enterococcus* spp., *Klebsiella* spp., and Escherichia coli, and the risk of SSI is significantly increased in the presence of bacterobilia [19,20,21]. However, the same meta-analyses failed to demonstrate a significant association between bacterobilia and POPF, overall morbidity, and mortality [19,20]. In our series, positive preoperative bile culture and high-risk polymicrobial flora indicators were not associated with major complications; in this regard, it appears consistent with studies reporting that bile colonization may be significant in terms of SSI, but cannot determine global major morbidity on its own.

Bile culture literature is also heterogeneous within itself. In some series, positive bile culture has been found to be associated with organ/space SSI and prolonged antibiotic treatment, while in others, bile culture has not been effective on overall complication rates, even though it guides antibiotic selection [22,23]. Specifically, in Groen et al.’s cohort and meta-analysis study, no significant association was found between intraoperative culture positivity and abdominal infectious complications. Even at the meta-analysis level, which included approximately 2000 patients, no significant increase in the risk of organ/space infection was demonstrated with positive culture [19]. In our analysis, bile culture was not an independent predictor of Clavien–Dindo ≥ III complications, even when detailed microbiological subclassifications such as Gram-negative or high-risk flora were included. Therefore, our study provides additional data supporting the literature’s position that bacterobilia may play a role in the full spectrum of SSI; however, it cannot explain major morbidity on its own.

To overcome the heterogeneity observed in previous bile culture studies and to go beyond the mere distinction between culture positivity/negativity, this study employed a hierarchical microbiological severity classification that incorporates both culture status and antibiotic resistance burden [11]. Patients who underwent bile culture were classified along a gradual spectrum of resistance ranging from no growth to pan-resistant colonization. Although numerical increases in major complications and infection-related outcomes were observed in higher microbiological severity categories, these differences did not reach statistical significance. This finding suggests that resistance burden alone may have limited prognostic value for major morbidity; its clinical relevance may instead be mediated through adequacy of antimicrobial coverage. Notably, even pan-resistant isolates did not constitute a distinct clinical phenotype in terms of major morbidity or complication severity in this cohort; however, this finding should be interpreted cautiously due to the limited number of PDR cases. As summarized in Table 5, patients with concomitant intra-abdominal culture positivity and XDR/PDR bile isolates did not show statistically significant differences in major complications, SSI, sepsis, or prolonged antibiotic use compared with the remaining cohort, despite a numerical gradient toward higher rates in patients with MDR/XDR/PDR flora.

In addition to resistance severity stratification, we performed a coverage-based assessment of prophylactic adequacy to determine whether antibiotic–pathogen mismatch influenced postoperative outcomes. Using a predefined algorithm incorporating antimicrobial class and resistance profiling, discordant prophylaxis was identified in 15.9% of patients. Major morbidity occurred in 30.4% of discordant cases compared with 18.0% of concordant cases (OR: 1.99; 95% CI: 0.69–5.36). After adjustment for age, ASA classification, and operative type, discordant prophylaxis remained associated with increased morbidity (adjusted OR: 1.84; 95% CI: 0.63–4.96). Although statistical significance was not reached, the magnitude of effect suggests a clinically relevant signal. These findings indicate that antimicrobial adequacy, rather than culture positivity alone, may represent a modifiable contributor to postoperative risk [24,25]. While previous studies have emphasized the dominant role of host-related risk factors in determining severe morbidity after PD, our findings suggest that antimicrobial adequacy may represent an additional, potentially modifiable contributor within this multifactorial framework [26].

Although data on intraoperative abdominal cultures and peritoneal contamination are more limited, significant studies have been conducted in this area in recent years. Sugiura et al. reported a positive culture rate of approximately 20% in abdominal fluid obtained after peritoneal lavage and showed that both incisional and organ/space SSI rates increased significantly in this case [3]. A more recent study has reported that intraoperative lavage fluid contamination is associated with the risk of organ/space infection after PD [27]. However, in most of these studies, the primary endpoint was POPF or SSI; Clavien–Dindo ≥ III general major morbidity was less frequently examined. In our cohort, intraoperative abdominal culture was not associated with significant complications even when Enterococcus, Pseudomonas/Acinetobacter, Gram-negative, and high-risk polymicrobial flora indicators were included; the integrated model combining bile and abdominal cultures yielded similar results. This suggests that although abdominal contamination may contribute to the spectrum of infectious complications after PD, it may play a relatively secondary role within the multifactorial clinical picture determining major morbidity.

In the adjusted clinical model, baseline variables including age, ASA classification, and operative type did not independently predict major morbidity. The limited discrimination of the core model (AUC 0.59) reflects the multifactorial nature of severe complications after PD and highlights the difficulty of risk stratification based solely on baseline clinical characteristics. Within this context, antimicrobial adequacy may represent an additional dimension of risk not captured by conventional clinical predictors. POPF, biliary fistula, and other technical complications are often related to gland texture, duct diameter, and surgical experience; however, the clinical impact of these complications is frequently modulated by the patient’s reserve and comorbidity profile [15,16,17]. Since our dataset lacked detailed intraoperative variables, such as pancreatic texture, duct diameter, operation time, and blood loss, we were unable to assess potential interactions between microbiological findings and these surgical risk factors. Nevertheless, even when bile and abdominal microbiological variables were incorporated into the model, overall discrimination remained limited. These findings suggest that culture-based risk stratification for predicting major morbidity is limited unless integrated with assessment of antimicrobial adequacy.

The clinical implications of these findings are important. The current literature suggests that preoperative biliary drainage may increase the risk of bacterobilia and surgical site infection (SSI); however, multicenter analyses show that the effect of PBD on overall morbidity and mortality remains controversial [7,28]. Although intraoperative culture-based antibiotic selection has been shown to reduce the risk of surgical site infections in some series, the routine application of this approach is debated in the context of broad-spectrum antibiotic exposure and antimicrobial resistance development [22,26]. In our cohort, culture positivity alone did not independently predict major morbidity; however, discordant prophylaxis showed a numerical increase in postoperative risk, although this difference did not reach statistical significance. These findings suggest that the primary value of bile culture may lie not in risk stratification based solely on positivity status, but in ensuring appropriate antimicrobial coverage. For this reason, major morbidity (Clavien–Dindo ≥ III) was selected as the primary endpoint because it represents clinically meaningful postoperative complications after pancreatoduodenectomy, while infection-specific outcomes such as surgical site infection, sepsis, and prolonged antibiotic therapy were analyzed as secondary endpoints. Accordingly, culture data should be interpreted within an antimicrobial stewardship framework rather than as standalone predictors of severe morbidity.

Previous systematic reviews and cohort studies have reported that preoperative bacterobilia and positive intraoperative bile culture may be associated with infectious or local complications, particularly surgical site infection (SSI), intra-abdominal infection, and biliary fistula [20,22,29]. However, several studies have failed to demonstrate a consistent association between bacterobilia and overall morbidity or mortality beyond SSI [30,31]. Our findings align with this latter perspective, indicating that culture positivity alone does not independently determine major morbidity severity. Within this context, antimicrobial adequacy rather than colonization status per se may represent the more clinically relevant variable.

Although preoperative bile microbiology has traditionally been evaluated as a prognostic marker, its clinical role in PTBD patients remains debated. Bacterobilia associated with PTBD is common, and culture positivity has been linked to infectious complications in some series [6,18]. Accordingly, there is an intuitive assumption that early initiation of broad-spectrum or targeted antibiotics following positive bile culture results may reduce postoperative complications. To explore this possibility, patients who underwent bile culture sampling were compared with those who did not; however, no significant differences were observed between these groups in terms of major postoperative complications (Clavien–Dindo ≥ III), complication severity, postoperative inflammatory markers, or length of hospital stay. The similarity in measured baseline characteristics between groups suggests that major observable differences were limited; however, residual selection bias related to clinician-directed culture acquisition cannot be completely excluded. Accordingly, our findings do not demonstrate an independent association between bile culture acquisition alone and severe postoperative morbidity after PD.

This study has particular strengths and limitations. A strength is that both preoperative bile and intraoperative abdominal cultures were systematically evaluated in a single-center, homogeneous patient group, and these cultures were categorized not only as positive/negative but also into clinically meaningful categories, such as Enterococcus, Pseudomonas/Acinetobacter, Gram-negative, Gram-positive, and “high-risk polymicrobial.” Furthermore, the assessment of major complications according to the Clavien–Dindo classification and the testing of bile/abdominal cultures in both univariate and multivariate models with potential confounders such as comorbidity, ASA, BMI, age, and type of operation increases the clinical interpretability of the results. However, the retrospective design, limited sample size, and low case numbers in some microbiological subgroups (e.g., at the specific pathogen level) increase the likelihood of type II errors, particularly in detecting small effect sizes. The perioperative antibiotic regimen and possible protocol changes were not modeled in detail, and known surgical risk factors such as pancreatic texture, duct diameter, operation time, and blood loss were not recorded. Therefore, the associations observed between microbiological variables and major morbidity should be interpreted cautiously, as residual confounding related to these unmeasured surgical risk factors cannot be excluded. Furthermore, since a systematic comparison could not be made in patients without abdominal and bile cultures, the group with cultures may carry selection bias. These points limit the generalizability of the findings and indicate the need for larger, prospective series, especially at the microbiological subtype level. Importantly, the relatively small number of patients receiving discordant prophylaxis (*n* = 23) limited statistical power to detect moderate effect sizes and increases the likelihood of type II error. The wide confidence intervals observed in multivariable analyses reflect limited precision rather than definitive absence of effect. Furthermore, the modest discrimination of the final model (AUC 0.63) suggests that additional clinical and intraoperative variables not captured in this dataset may contribute to postoperative risk.

## 5. Conclusions

In this retrospective PTBD cohort undergoing pancreatoduodenectomy, preoperative bile culture positivity and resistance severity were not independently associated with major postoperative morbidity. Discordant perioperative antibiotic prophylaxis showed a numerical increase in major complication rates; however, this observation did not reach statistical significance and should be interpreted cautiously given the limited sample size and wide confidence intervals. These findings suggest that the primary clinical value of bile culture may lie in ensuring adequate antimicrobial coverage rather than in risk stratification based solely on colonization status. While baseline clinical factors showed limited predictive performance in our model, antimicrobial adequacy may represent a potentially modifiable contributor within the multifactorial framework of postoperative morbidity. Larger prospective studies are warranted to determine whether discordant prophylaxis independently influences outcomes in adequately powered cohorts.

## Figures and Tables

**Table 1:** Baseline characteristics of the study population.

Variable	Value
Number of patients, *n*	145
Age, years, median (IQR)	63 (57–70)
BMI, kg/m^2^, median (IQR)	25.0 (22.8–28.4)
Sex, *n* (%)	Male: 83 (57.2%), Female: 62 (42.7%)
ASA score, *n* (%)	I: 7 (4.8%), II: 74 (51%), III: 59 (40.4%), IV: 5 (3.4%)
Comorbidity burden, median (IQR)	1 (0–2)
Surgical procedure, *n* (%)	Classical Whipple: 114 (78.6%), Pylorus-preserving Whipple: 31 (21.3%)
Major complications, *n* (%)	29/145 (20%)
Length of hospital stay, days, median (IQR)	14 (9–23)

This table summarizes demographic, anthropometric and perioperative clinical features of all patients undergoing pancreaticoduodenectomy. Continuous variables are presented as median (interquartile range) and categorical variables as counts and percentages. Missing values were reported where applicable. Major postoperative complications were defined as Clavien–Dindo grade ≥ III and were calculated across the entire cohort (*n* = 145).

**Table 2:** Postoperative outcomes according to preoperative bile culture status.

Variable	No Culture (*n* = 30)	Culture-Negative (*n* = 59)	Culture-Positive (*n* = 56)	*p*-Value
BMI	25.4 (22.6–32.1)	25.7 (22.6–27.5)	25.0 (23.1–28.9)	0.506
Length of stay (days)	14.5 (9.0–25.8)	13.0 (9.0–22.0)	15.0 (9.8–21.5)	0.819
POD4 WBC	9870 (7685–11,708)	8070 (7060–11,210)	8230 (6623–10,295)	0.300
POD4 CRP	160.4 (72.6–220.8)	110.3 (68.8–176.1)	143.2 (77.9–204.8)	0.278
Major complication (CD ≥ III)	8 (26.7%)	9 (15.3%)	12 (21.4%)	0.406
Prolonged antibiotic therapy	5 (16.7%)	12 (20.3%)	14 (25.0%)	0.678
Surgical site infection	1 (3.3%)	3 (5.1%)	4 (7.1%)	0.806
Sepsis	1 (3.3%)	1 (1.7%)	1 (1.8%)	1.000

Patients were grouped as no culture obtained, culture-negative, or culture-positive based on preoperative bile culture results. Major complications were defined as Clavien–Dindo grade ≥ III. Continuous variables are presented as medians (interquartile range) and compared using the Kruskal–Wallis test; categorical variables are presented as *n* (%) and compared using Fisher’s exact test. Postoperative day 4 white blood cell count (WBC) and C-reactive protein (CRP) were analyzed using available data. A two-sided *p*-value < 0.05 was considered statistically significant.

**Table 3:** Postoperative outcomes according to intra-abdominal culture status.

Outcome	No Culture (*n* = 52)	Culture-Negative (*n* = 30)	Culture-Positive (*n* = 63)	*p*-Value
Major complications (CD ≥ III), n (%)	7 (13.5)	6 (20.0)	16 (25.4)	0.281
Surgical site infection, n (%)	2 (3.8)	3 (10.0)	3 (4.8)	0.510
Prolonged antibiotic therapy, n (%)	6 (11.5)	5 (16.7)	20 (31.7)	**0.026**
POD4 WBC, median (IQR)	8350 (7035–10,265)	8185 (6492–11,178)	8425 (6990–10,792)	0.990
POD4 CRP, median (IQR)	118.5 (69.5–187.4)	111.9 (70.4–174.0)	150.3 (75.8–222.1)	0.263

Patients were classified into three groups based on intra-abdominal culture records: no culture obtained, culture obtained with no growth, and culture positive. Major complications were defined as Clavien–Dindo grade ≥ III. Continuous variables are presented as median (Q1–Q3) and compared using the Kruskal–Wallis test; categorical variables are presented as *n* (%) and compared using Fisher’s exact test.

**Table 4:** Postoperative outcomes according to microbiological severity of bile cultures.

Microbiological Severity	*n*	Major Complication *n* (%)	SSI *n* (%)	Sepsis *n* (%)	Prolonged Antibiotic Therapy *n* (%)
No growth	59	9 (15.3)	3 (5.1)	1 (1.7)	12 (20.3)
Low resistance	23	4 (17.4)	1 (4.3)	1 (4.3)	6 (26.1)
High resistance (MDR/XDR/PDR)	33	8 (24.2)	3 (9.1)	0 (0.0)	8 (24.2)
*p*-value	—	0.77	0.77	0.92	0.81

Microbiological severity was classified as no growth, low resistance (non-MDR isolates), and high resistance (MDR or PDR isolates) based on bile culture results. Major complications were defined as Clavien–Dindo grade ≥ III. Categorical variables were compared using Fisher’s exact test. A two-sided *p*-value < 0.05 was considered statistically significant.

**Table 5:** Sensitivity analysis in patients with intra-abdominal culture positivity and XDR/PDR bile isolates.

Outcome	Others (*n* = 134)	Abdominal Culture Positivity (XDR/PDR) (*n* = 11)	*p*-Value
Major complications (CD ≥ III)	27 (20.1%)	2 (18.2%)	1.000
Prolonged antibiotic therapy	30 (22.4%)	3 (27.3%)	0.458
POD4 WBC, median (Q1–Q3)	8300 (6898–10,535)	9190 (6610–10,605)	0.862
POD4 CRP, median (Q1–Q3)	126.4 (69.5–206.0)	148.6 (111.7–213.4)	0.234

This analysis evaluates whether the coexistence of intra-abdominal culture positivity and high-level antimicrobial resistance (XDR/PDR) modifies postoperative outcomes. Patients meeting both criteria (*n* = 11) were compared with all remaining patients (*n* = 134). Major complications were defined as Clavien–Dindo grade ≥ III. Continuous variables are presented as median (Q1–Q3) and were compared using the Wilcoxon rank-sum test. Categorical variables were analyzed using Fisher’s exact test.

**Table 6:** Coverage-Based Prophylaxis Adequacy and Postoperative Outcomes.

Outcome	Concordant Coverage (*n* = 122)	Discordant Coverage (*n* = 23)	OR (95% CI)	*p*-Value
Major complication (≥III)	22 (18.0%)	7 (30.4%)	1.99 (0.69–5.36)	0.25

Prophylactic adequacy was defined using a predefined coverage-based algorithm incorporating antimicrobial class and resistance profiling. Discordant prophylaxis was defined as lack of in vitro coverage against at least one clinically significant isolate identified in preoperative PTBD bile culture. Major complications were defined as Clavien–Dindo grade ≥ III. Odds ratios (OR) with 95% confidence intervals (CI) were calculated using Fisher’s exact test.

## Data Availability

No new data were created or analyzed in this study.
